# Immune dysregulation in *Pneumocystis jirovecii* infection and the development of a machine learning-based diagnostic model

**DOI:** 10.3389/fimmu.2026.1795306

**Published:** 2026-09-08

**Authors:** Jianchao Wu, Yue Ning, Xing Li, Sheng Wang, Juan Chen, Meixia Zheng, Jin Zhu, Jun Lu

**Affiliations:** The Quzhou Affiliated Hospital of Wenzhou Medical University, Quzhou People’s Hospital, Quzhou, China

**Keywords:** diagnosis, immune response, *P. jirovecii* colonization, *Pneumocystis jirovecii* pneumonia, random forest model

## Abstract

**Background:**

*Pneumocystis jirovecii* pneumonia (PJP) is a life-threatening opportunistic infection in immunocompromised patients. A major clinical challenge is the inability of current microbiological diagnostics (e.g., quantitative polymerase chain reaction [qPCR]) to reliably distinguish PJP from *Pneumocystis jirovecii* colonization(*P. jirovecii* colonization).

**Objective:**

This study aimed to develop and validate a diagnostic model for differentiating PJP from *P. jirovecii* colonization by analyzing 17 core model input predictors that integrate pathogen burden and host immune response profiles.

**Methods:**

We conducted a retrospective analysis of 127 clinically assessed adult patients (78 with PJP, 49 with *P. jirovecii* colonization). 17 core model input predictors (pathogen burden, systemic inflammatory markers, and peripheral blood cellular immune parameters) were compared between groups. Subsequently, we constructed a random forest machine learning model using these 17 core model input predictors. Model development employed a two-stage grid search strategy for hyperparameter optimization, with strict prevention of overfitting ensured through cross-validation. The final model’s performance was evaluated on an independent internal validation set.

**Results:**

Compared to the *P. jirovecii* colonization group, the PJP group exhibited significantly higher pathogen burden (median Ct 31.40 vs. 34.72, *P<*0.001), heightened inflammatory responses (e.g., elevated IL-10 and IL-6), and a characteristic immunosuppressed state marked by severe lymphopenia, with pronounced reductions in CD4^+^ T cells and regulatory T cells (Tregs). Based on these findings, a Random Forest algorithm was used to construct a discriminative model incorporating the 17 core model input predictors. In the validation cohort (N = 37), the model demonstrated robust discriminatory performance, with an area under the ROC curve (AUC) of 0.852 (95% CI: 0.709–0.996) and an accuracy of 78.38%. Feature importance analysis identified three top core model input predictors: pathogen load, IL-10 level, and absolute lymphocyte count as the most discriminative predictors. This study confirms that a multi-parameter strategy integrating the 17 core model input predictors can effectively overcome the limitations of current single-marker microbiological tests. The developed machine learning model provides a novel, translationally promising tool for the precise clinical differentiation of PJP from *P. jirovecii* colonization.

## 1 Introduction

*Pneumocystis jirovecii* is an obligate fungal pathogen specific to humans (1, 2)immunocompetent individuals, it often exists as an asymptomatic colonizer of the alveoli. However, in hosts with significantly reduced or dysfunctional CD4^+^ T cells, it can cause fatal *Pneumocystis jirovecii* pneumonia (PJP) (3, 4). With the advancement of Human Immunodeficiency Virus (HIV) prevention and control systems, non HIV immuno compromised populations—such as organ transplant recipients and patients with auto immune diseases—have become the primary groups affected by PJP. The disease carries a high mortality rate of 30–40% in these patients, posing a severe clinical challenge (5, 6).

Current diagnosis relies on imaging and microbiological tests. Although highly sensitive, chest highresolution computed tomography (HRCT) lacks specificity, while traditional microscopy-based methods suffer from inadequate sensitivity (7, 8). Molecular detection techniques, such as quantitative polymerase chain reaction (qPCR), can detect pathogen nucleic acids with high sensitivity but fail to reliably differentiate active infection from asymptomatic *P. jirovecii* colonization. This fundamental limitation often complicates clinical decision-making, potentially leading to unnecessary treatment or therapeutic delays (9).

Studies have indicated that *P.jirovecii* infection triggers ahostimmune response, inwhichTcell-mediated specific immunity plays a central role in controlling the infection. Depletion of CD4^+^ T cells is a key risk factor for developing disease, while distinct functional subsets of CD8^+^ T cells may play opposing roles in defense versus lung tissue injury (10, 11). In active infection, the host mounts a pronounced inflammatory response characterized by elevated pro-inflammatory cytokines (IFN-*γ*, IL-6) that contribute to pulmonary inflammation and tissue damage, alongside a compensatory increase in anti-inflammatory IL-10 that may paradoxically suppress protective T-cell immunity and promote immune dysfunction. Concurrently, severe peripheral lymphopenia—particularly reductions in absolute lymphocyte counts—reflects the impaired cellular immune capacity that facilitates transition from colonization to overt pneumonia (12, 13). Therefore, a comprehensive profiling integrating pathogen burden (qPCR Ct value), systemic inflammatory mediators (cytokines and acute-phase reactants), and peripheral blood cellular immune parameters may provide a more complete picture of the host–pathogen interaction.

To address the critical limitation of current microbiological diagnostics (e.g., qPCR) in distinguishing PJP from asymptomatic *P. jirovecii* colonization, this study aims to achieve three core objectives: first, to systematically profile the host immune responses in both groups by analyzing systemic inflammatory markers (including a panel of cytokines), peripheral blood immune cell subsets (e.g., CD4^+^ T cells, regulatory T cells), and lymphocyte populations; second, to compare the pathogen burden (assessed by qPCR cycle threshold [Ct] values) between PJP patients and *P. jirovecii* colonized individuals; and third, to integrate the aforementioned pathogen-derived and host-derived data to develop and validate a machine learning-based diagnostic model (using the Random Forest algorithm) for precise differentiation of PJP from *P. jirovecii* colonization. By integrating these multi-dimensional datasets, we seek to provide a novel, clinically translatable tool that overcomes the shortcomings of single-marker tests and improves diagnostic decision-making for immunocompromised patients at risk of PJP.

## 2 Methods

### 2.1 Study design and settings

This retrospective cohort study included adult inpatients (⩾18 years old) with clinically suspected PJP at Quzhou Hospital of Wenzhou Medical University between July 2024 and October 2025, who tested positive for *P. jirovecii* via qPCR in bronchoalveolar lavage fluid or sputum samples. According to the guidelines for invasive fungal infections (14), a definitive diagnosis of PJP requires direct visualization of *P. jirovecii* cysts or trophozoites in bronchoalveolar lavage fluid (BALF), sputum, or lung biopsy specimens via conventional or immunofluorescence staining. However, these staining methods are not routinely available at our institution, which necessitated the formulation of alternative composite diagnostic criteria for stratifying PJP and *P. jirovecii* colonization in this study.

The study protocol was approved by the Ethics Committee Approval Letter of Biomedical Research Involving Humans (Approval No. 2026-161), which waived the requirement for written informed consent because of the retrospective nature of the study.

### 2.2 Data collection

The following demographic and clinical data were collected from patient medical records: (1) Basic information (age, sex, smoking history, alcohol consumption history); (2) Past medical history (underlying comorbidities, history of infectious diseases and malignancies); (3) The 17 core model input predictors for subsequent random forest model construction: pathogen burden (qPCR Ct values), systemic inflammatory markers (white blood cell count, lymphocyte count, neutrophil count, high-sensitivity C-reactive protein, a panel of seven cytokines, lactate dehydrogenase), and peripheral blood cellular immune parameters; (4) History of medication use, 1,3-*β*-D-glucan level, chest CT scan findings, and mNGS data (3 cases).

Two experienced clinical microbiologists independently adjudicated all cases as either PJP infection or *P. jirovecii* colonization based on a composite of clinical features, laboratory results (including 1,3*β*-D-glucan levels), chest CT findings, the qualitative qPCR result (positive/negative), and mNGS data, as summarized in **Table 1**. The adjudicating experts were blinded to the qPCR cycle threshold (Ct) values. Following diagnostic classification, the corresponding Ct values were retrospectively retrieved from laboratory records to serve as a continuous variable representing pathogen burden in subsequent analytical modeling.

**Table 1:** Eligibility criteria of the 2 different groups included in the study.

Criteria	PJP group	*P. jirovecii* colonization group
Inclusion criteria	1. Presence of respiratory symptoms: at least one of the following: cough, dyspnea, pleuritic pain, hemoptysis) or fever ⩾ 37.8 °C.	1. Positive results from qPCR or mNGS.
	2. Characteristic chest CT findings: diffuse ground-glass opacity around bilateral hilar regions, accompanied by interstitial infiltration.	2. Confirm alternative causes of acutelung infection, and the radiological features are inconsistent with PJP.
	3. Positive results from qPCR or mNGS	3. The results of 1,3-*β*-D-glucan detection are continuously negative.
Exclusion criteria	Patients who received PJP-related drug therapy (e.g., Trimethoprim Sulfamethoxazole, clindamycin, primaquine, atovaquone) within 5 days prior to qPCR testing	

This table summarizes the inclusion and exclusion criteria for the PJP group and the *P. jirovecii* colonization group. PJP, *P. jirovecii* pneumonia; qPCR, quantitative polymerase chain reaction; mNGS, metagenomic next-generation sequencing.

Data for exploratory in-depth immunological analyses (comprehensive immune cell subset analysis and regulatory T-cell [Treg] functional analysis) were obtained from clinical records. These specialized assays are not part of routine workup for suspected PJP and were ordered at the discretion of the treating physician based on specific clinical indications during patient care. Consequently, these data were available only for the subset of patients who underwent such testing as part of their clinical management, resulting in the subcohorts for Treg analysis (N = 41) and immune cell subset analysis (N = 24). Notably, these exploratory immunological data were not included in the 17 core model input predictors and had no involvement in random forest model development.

### 2.3 Blood sample processing and laboratory assays

#### 2.3.1 Sample collection

Peripheral blood samples (5 mL per tube) were collected during routine clinical care using two types of vacuum tubes: K_2_-ethylenediaminetetraacetic acid (EDTA) tubes for complete blood count, cytokine profiling and immunophenotyping, and serum separator tubes (yellow-top) for biochemical analysis. All samples for the assays described below were obtained within a five-day window before or after the diagnostic qPCR test of respiratory specimens.

#### 2.3.2 Sample processing

For biochemical analysis, blood collected in serum separator tubes was centrifuged following a standard protocol, and the resulting serum was used for batched analysis. For complete blood count (CBC) with differential, cytokine measurement and immunophenotyping, fresh whole blood from EDTA tubes was used directly.

#### 2.3.3 Laboratory assays

Systemic inflammatory markers. Serum levels of high-sensitivity C-reactive protein (hs-CRP) and lactate dehydrogenase (LDH) were measured on an Abbott Alinity ci analyser.

Cytokine profiling. Levels of seven cytokines (IL-2, IL-4, IL-6, IL-10, IL-17A, TNF-*α*, IFN-*γ*) were quantified in EDTA plasma using a commercial multiplex cytokine assay kit (Qingdao Raisecare Bio-technology Co., Ltd.) on a DxFLEX flow cytometer (Beckman Coulter, USA).

Immunophenotyping. For the designated cell subsets, exploratory immune cell subset analysis and regulatory T-cell (Treg, defined as CD4^+^CD25^+^CD127lowFoxP3^+^)quantification were performed on fresh EDTA whole blood using specific human lymphocyte subset and Treg detection kits (Beijing Tongsheng Shidai Biotechnology Co., Ltd.) on the DxFLEX flow cytometer.

The standardized Treg staining protocol was performed per kit instructions: 1) Surface staining: 50 *µ*L fresh EDTA peripheral blood was incubated with CD4-FITC, CD25-PE, CD127-PerCP-Cy5.5 antibodies at room temperature in dark for 15 min. 2) Lysis & fixation: Red blood cell lysis buffer was added, followed by washing and formaldehyde fixation. 3) Intracellular FoxP3 staining: Cells were permeabilized, stained with FoxP3-APC, and incubated in dark for 30 min. 4) Data acquisition & analysis: 10,000 lymphocyte events were collected. Tregs were gated as CD4^+^CD25^+^CD127lowFoxP3^+^ populations to calculate Treg absolute count and percentage.

Complete blood count (CBC). White blood cell(WBC), absolute lymphocyte, monocyte and neutrophil counts wereperformed onfresh EDTAwhole bloodusingautomated haematologyanalysers. Retrospective data abstraction. All laboratory values, including those from the specialized assays described above, were retrospectively extracted from the hospital’s electronic medical record and laboratory information systems.

### 2.4 Quantitative real time PCR for *P. jirovecii* detection

*P. jirovecii* nucleic acid was detected and quantified using the commercial “Aspergillus spp., Cryptococcus neoformans, and *P. jirovecii* Nucleic Acid Detection Kit (qPCR Fluorescent Probe Method)” (ABT Zhuocheng Huisheng Bioscience, Beijing, China), according to the manufacturer’s instructions. Briefly, DNA was extracted from respiratory specimens. qPCR was performed on an ABI 7500 Real Time qPCR System (Applied Biosystems) targeting the mitochondrial large subunit ribosomal RNA (mtLSU rRNA) gene of *P. jirovecii*, with the CY5 channel used for fluorescence detection.

The cycle threshold (Ct) value, representing the qPCR cycle at which the fluorescent signal exceeded a pre defined threshold, was recorded for each sample. In this study, the Ct value was used as a relative, inverse correlate of fungal DNA load (i.e., a lower Ct value indicates a higher pathogen burden). It should be noted that the commercial kit is calibrated and intended for qualitative diagnosis (positive/negative) using a defined cut off (Ct ≤ 36 for a positive result, as per the kit instructions) and does not provide absolute quantification (e.g., gene copies per millilitre) via a standard curve. Therefore, the Ct value served as a semi quantitative, continuous variable in the analytical models, reflecting the relative abundance of pathogen DNA.

### 2.5 Statistical analysis

All statistical analyses were performed using R software (v4.2.1; R Foundation for Statistical Computing, Vienna, Austria). For continuous variables, the Shapiro–Wilk test was used to assess the normality of distribution, and the Levene test to evaluate the homogeneity of variances. Normally distributed data are presented as mean ± standard deviation (s.d.), with group comparisons conducted using the two-sample ttest (for two groups) or one-way analysis of variance(ANOVA; forthree or moregroups); *post-hoc*pairwise comparisons were performed with the Tukey’s honestly significant difference (HSD) test. Non-normally distributed data are reported as median (interquartile range, IQR), with group comparisons carried out using the Mann–Whitney Utest(fortwogroups) or Kruskal–Wallistest (for three ormore groups); *post-hoc* analyses were implemented with the Bonferroni-corrected Dunn’s test. Categorical variables are presented as n (%), with group comparisons made using Pearson’s chi-squared test or Fisher’s exact test (when expected cell frequencies were <5). The Cochran–Armitage trend test was applied for ordinal categorical variables. All tests were two-tailed, and a *P<*0.05 was deemed statistically significant.

### 2.6 Construction of the random forest model

Based on the clinical and pathophysiological mechanisms of PJP, we selected 17 core model input predictors obtainable at the time of suspected diagnosis, including pathogen load (qPCR cycle threshold, Ct value), 8 systemic inflammatory markers and 8 cellular immune parameters. All 17 core model input predictors were derived from the full core cohort of 127 patients (78 with PJP, 49 with *P. jirovecii* colonization), and no univariate prescreening was performed for these predictors to avoid selection bias. A total of 127 patients were randomly assigned to a training set and an internal validation set; all model construction steps were conducted exclusively on the training set, with the validation set used independently for the final model evaluation.

A random forest classification model was built using the randomForest package in R Studio. A baseline model was first established with default parameters: for the classification task, the number of candidate variables randomly selected at each node splitting (mtry) was set to the default value of $\sqrt{p}$ (where p represents the total number of candidate predictors), and the number of decision trees (ntree) was set to the default of 500. Hyperparameters (mtry and ntree) were subsequently optimized through 5-fold cross-validation coupled with a two-stage grid search.

Variable importance was quantified by the mean decrease in the Gini coefficient, with higher values indicating a greater contribution of the corresponding variable to model classification. Model performance was evaluated using the area under the receiver operating characteristic curve (AUC), accuracy, sensitivity, specificity, positive predictive value (PPV), negative predictive value (NPV), F1 score and calibration. Overfitting and generalization ability were determined by comparing performance metrics between the training set and the validation set. This work provides a methodological basis for the accurate differentiation between PJP and *P. jirovecii* colonization.

## 3 Results

### 3.1 Characteristics of the study population

**Figure 1:**
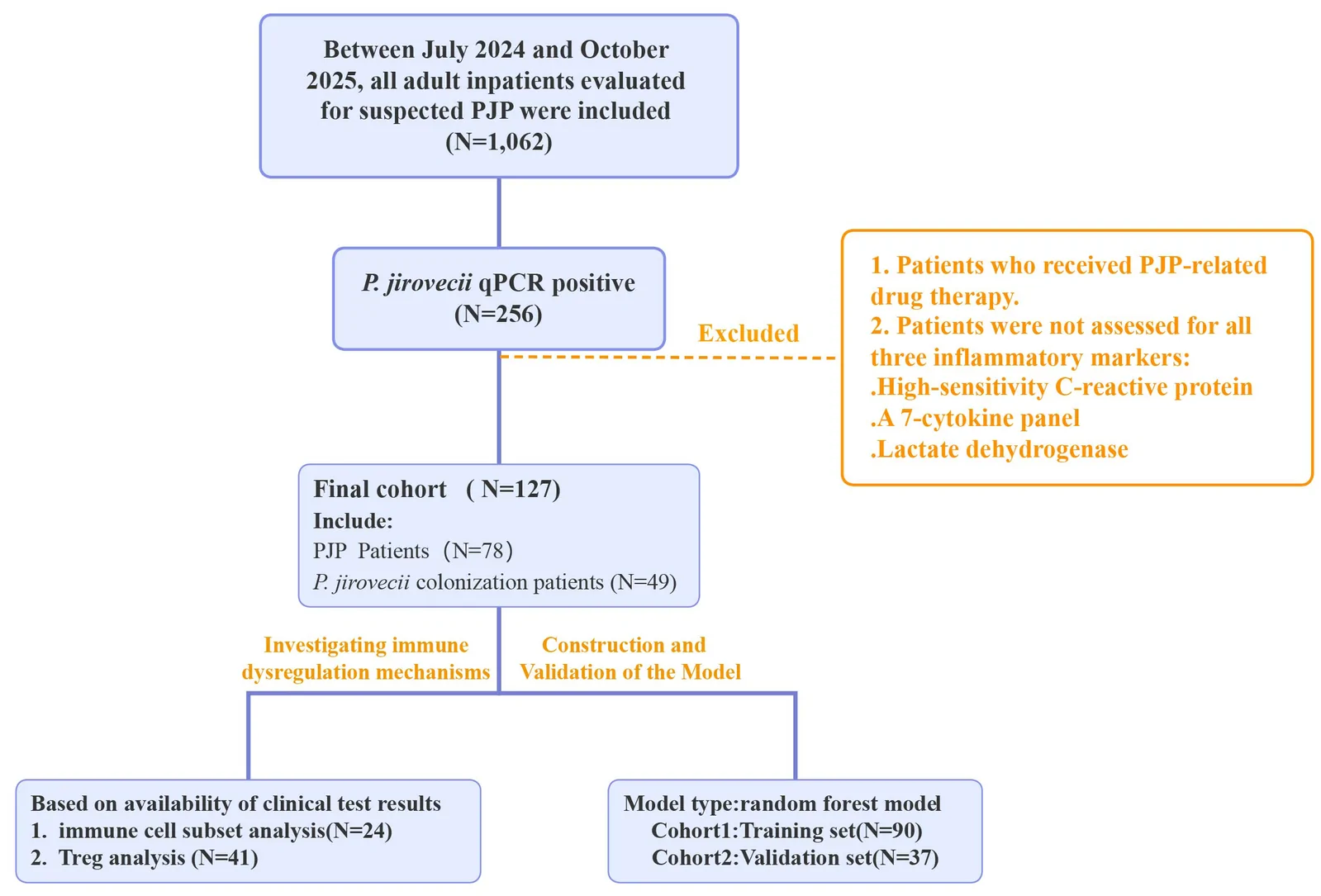
Between July 2024 and October 2025, 1,062 adult inpatients evaluated for suspected PJP were included; 256 tested positive for *P. jirovecii* by qPCR, and patients were excluded if they received PJP-related drug therapy or were not assessed for all three required inflammatory markers (high-sensitivity C-reactive protein, 7-cytokine panel, lactate dehydrogenase), resulting in a final cohort of 127 patients (78 with PJP, 49 with *P. jirovecii* colonization), from which two parallel analyses were conducted: immune dysregulation investigation via subset analyses of immune cell subsets (N = 24) and regulatory T cells (Treg, N = 41) based on clinical test availability, and random survival forest model construction and validation using a training set (N = 90) and independent validation set (N = 37).

During the study period, 256 patients tested positive by qPCR. Of these, 127 patients (78 with PJP and 49 with *P. jirovecii* colonization) met the inclusion criteria and had complete data for the 17 core model input predictors, forming the full core cohort for the analysis of the 17 core model input predictors and subsequent random forest model construction. Based on the retrospective availability of specialized test results in the clinical records, 41 patients from this core cohort (26 PJP and 15 P*. jirovecii* colonization) had undergone regulatory T cell functional analysis, and 24 patients (17 PJP and 7 P*. jirovecii* colonization) had undergone comprehensive immune cell subset phenotyping in **Figure 1**. All blood samples used for the aforementioned assays were collected within a five day window before or after the qPCR testing.

The demographic and clinical characteristics of the 127 patients, stratified into the PJP group and the *P. jirovecii* colonization group, are presented in **Table 2**. No statistically significant differences were observed in baseline demographic features between the two groups. The overall mean age was 72 years (71 years in the PJP group vs. 73 years in the *P.jirovecii* colonizationgroup). Males constituted over 84% of both groups. The distribution of smoking history (51.28% vs. 51.02%) and alcohol consumption history (32.05% vs. 30.61%) was also similar. Glucocorticoid use was comparable between groups: 29 (37.18%) in the PJP group vs. 23 (46.94%) in the P. jirovecii colonization group. Regarding the spectrum of underlying comorbidities, no significant differences were found between the groups. The most common comorbidity was chronic obstructive pulmonary disease (COPD) (37.18% in PJP vs. 46.94% in *P. jirovecii* colonization), followed by hypertension (38.46% vs. 36.73%), with no statistically significant differences observed between the two groups for either condition (*P>*0.05, non-significant). The prevalence of diabetes mellitus (17.95% vs. 20.41%), chronic kidney disease (12.82% vs. 10.20%), and autoimmune diseases (6.33% vs. 4.10%) was also comparable between groups *P>*0.05, non-significant). A history of tuberculosis was reported in 8.97% of the PJP group and 12.24% of the *P. jirovecii* colonization group, with no significant intergroup difference (*P>*0.05, non-significant). Concerning malignancies, the PJP group had a numerically higher overall prevalence of hematological malignancies (14.10% vs. 10.20%), lung malignancies (11.54% vs. 10.20%), and other solid tumors (20.51% vs. 6.12%) compared to the *P. jirovecii* colonization group, though these differences did not reach statistical significance (*P>*0.05, non-significant). In terms of prognosis, the 30-day mortality rate was numerically higher in the PJP group (14.10%) than in the *P. jirovecii* colonization group (6.12%), with no significant intergroup difference (*P>*0.05, non-significant).

**Table 2:** Demographics and clinical characteristics of PJP and *P. jirovecii* colonization patients.

Category	Variables	PJP (N = 78)	*P. jirovecii* colonization (N = 49)	*P* value
Characteristics	Age (years)	71	73	0.15
	Male	66 (84.62%)	42 (85.71%)	1
	Smoking history (⩾ 5 years)	40 (51.28%)	25 (51.02%)	1
	Drinking history (⩾ 5 years)	25 (32.05%)	15 (30.61%)	1
	Glucocorticoid use	29 (37.18%)	23 (46.94%)	0.28
Comorbidities	Hypertension	30 (38.46%)	18 (36.73%)	0.99
	Diabetes mellitus	14 (17.95%)	10 (20.41%)	0.91
	Chronic kidney disease	10 (12.82%)	5 (10.20%)	0.87
	COPD	29 (37.18%)	23 (46.94%)	0.28
	Autoimmune disease	5 (6.33%)	2 (4.10%)	0.87
	Tuberculosis	7 (8.97%)	6 (12.24%)	0.77
	Hematological malignancies	11 (14.10%)	5 (10.20%)	0.71
	Lung cancer	9 (11.54%)	5 (10.20%)	1
	Other solid tumors(besides from lung cancer)	16 (20.51%)	3 (6.12%)	0.09
Mortality rate	30-day mortality rate	11 (14.10%)	3 (6.12%)	0.52

PJP refers to Pneumocystis jirovecii pneumonia; COPD is short for chronic obstructive pulmonary disease; 30-day mortality rate refers to the mortality outcome within 30 days after the relevant study time point. Glucocorticoid use was defined as prednisone equivalent dose ⩾20 mg administered continuously for at least 3 days within 60 days of admission.

### 3.2 Comparison of the 17 core model input predictors

**Figure 2:**
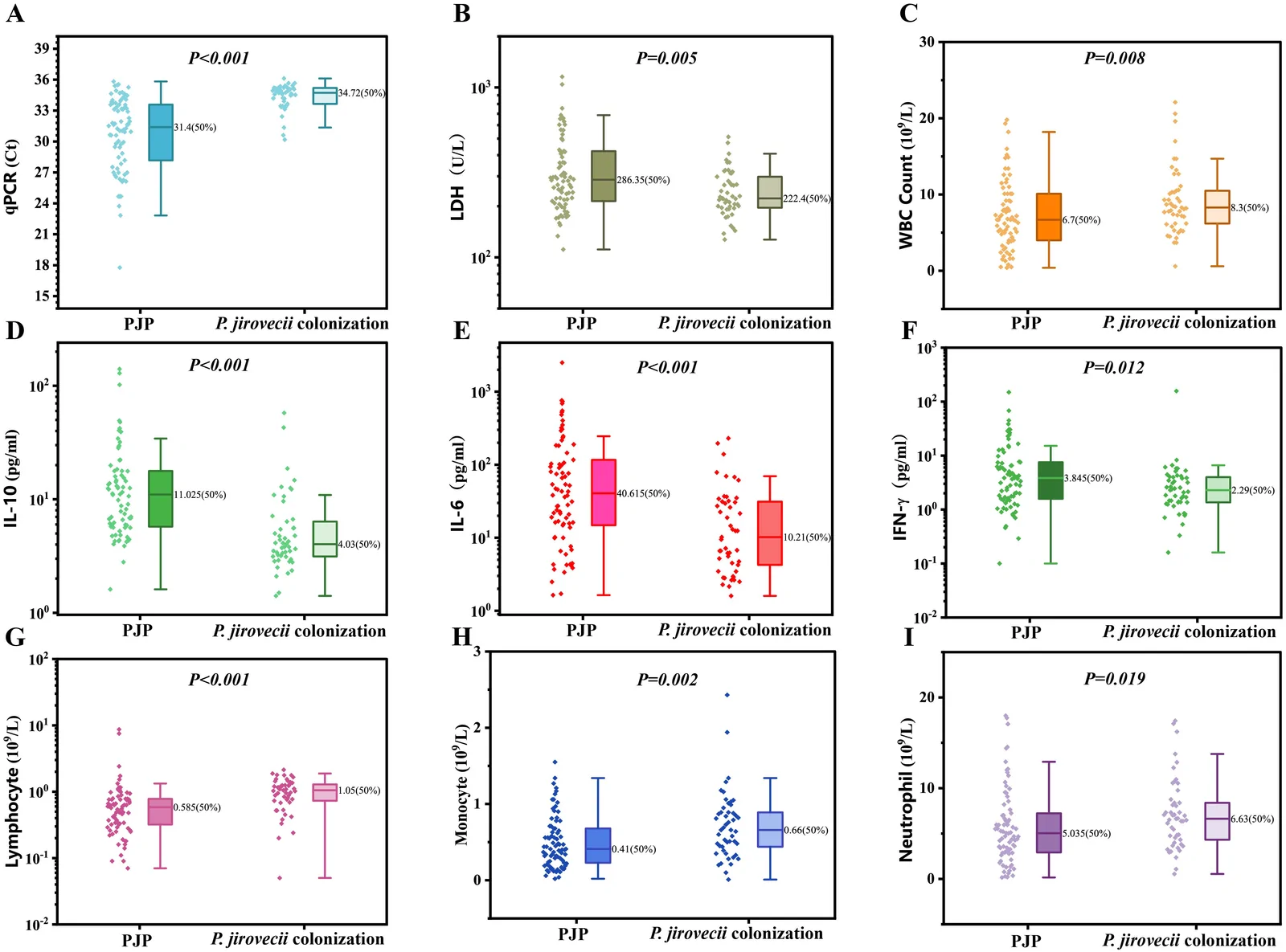
Box plots of laboratory indices with statistically significant differences between the PJP group and *P. jirovecii* colonization group. Panels correspond to the following indices: **(A)** qPCR(Ct); **(B)** LDH (U/L); **(C)** WBC count (10^9^/L); **(D)** IL-10 (pg/mL); **(E)** IL-6 (pg/mL); **(F)** IFN-*γ* (pg/mL); **(G)** Lymphocyte count (10^9^/L); **(H)** Monocyte count (10^9^/L); **(I)** Neutrophil count (10^9^/L). PJP, *P. jirovecii* pneumonia; qPCR(Ct), polymerase chain reaction cycle threshold; LDH, lactate dehydrogenase; WBC, white blood cell; IL, Interleukin; IFN-*γ*, interferon-*γ*.

Univariate analysis revealed distinct profiles of pathogen burden, host inflammatory response, and leukocyte counts between patients with PJP and those with *P. jirovecii* colonization in **Table 3**. The PJP group demonstrated a significantly higher fungal load, as indicated by lower qPCR Ct values (median 31.40 vs. 34.72, *P<*0.001), and elevated levels of the inflammatory markers LDH and IL-6. A pronounced anti-inflammatory or immunoregulatory response was also evident, characterized by a substantial increase in IL-10 levels (11.03 vs. 4.03 pg/mL, *P<*0.001). Hematologically, PJP patients exhibited significant leukopenia, driven primarily by profound reductions in absolute counts of lymphocytes, monocytes, and neutrophils. Notably, absolute lymphocyte count was markedly lower in the PJP group(0.58vs. 1.05×10^9^/L, *P<*0.001). No significant intergroup differences were found for hs-CRP, IL-2, IL-17A, TNF-*α*, IL-4, or the differential percentages of leukocyte subsets (all *P>*0.05, in **Table 3**). Notably, leukocyte percentage parameters exhibited far weaker predictive capacity than absolute cell counts, and only served as auxiliary indicators; absolute immune cell counts functioned as the core markers to distinguish active PJP from asymptomatic colonization. The laboratory indices showing statistically significant between-group differences are illustrated in **Figure 2**.

**Table 3:** Univariate analysis of laboratory parameters in PJP and *P. jirovecii* colonization patients.

Variables	PJP (N = 78)	*P. jirovecii* colonization (N = 49)	P value	Sig.
qPCR (Ct)	31.40 (28.25-33.58)	34.72 (33.65-35.19)	*<*0.001	***
LDH (U/L;NR:100-240)	286.35 (215.35-421.55)	222.40 (196.00-298.40)	0.005	**
Hs-CRP (mg/L;NR:0-10)	45.64 (19.34-94.25)	35.59 (18.14-75.95)	0.371	NS
IL-10 (pg/mL;NR:0-4.91)	11.03 (5.78-17.75)	4.03 (3.14-6.37)	*<*0.001	***
IL-6 (pg/mL;NR:0-5.30)	40.62 (15.01-114.10)	10.21 (4.25-31.24)	*<*0.001	***
IFN-*γ*(pg/mL;NR:0-7.42)	3.84 (1.59-7.53)	2.29 (1.36-3.99)	0.012	*
IL-2(pg/mL;NR:0-5.71)	0.84 (0.65-1.33)	0.98 (0.50-1.99)	0.776	NS
IL-17A (pg/mL;NR:0-20.60)	1.88 (0.99-5.50)	2.27 (1.36-4.25)	0.619	NS
TNF-*α* (pg/mL;NR:0-4.60)	1.77 (0.89-2.89)	1.97 (0.62-2.85)	0.430	NS
IL-4 (pg/mL;NR:0-4.91)	1.18 (0.71-1.72)	1.28 (0.63-2.04)	0.505	NS
WBC count (109/L;NR:4.00-10.0)	6.65 (4.10-9.88)	8.30 (6.20-10.50)	0.008	**
Lymphocyte (109/L;NR:0.80-4.00)	0.58 (0.32-0.78)	1.05 (0.73-1.29)	*<*0.001	***
Monocyte (109/L;NR:0.00-1.20)	0.41 (0.23-0.68)	0.66 (0.44-0.89)	0.002	**
Neutrophil (109/L;NR:2.00-7.00)	5.04 (2.93-7.17)	6.63 (4.32-8.39)	0.019	*
Lymphocyte (%)(NR:20.0-40.0)	8.65 (5.43-16.05)	10.90 (7.40-16.90)	0.179	NS
Monocyte (%)(NR: 0-12)	7.20 (5.10-10.15)	8.20 (6.20-10.20)	0.403	NS
Neutrophil (%)(NR:50.0-70.0)	80.85 (71.38-87.95)	76.90 (69.60-83.30)	0.248	NS

In this table, continuous variables (expressed as median (interquartile range, 25th–75th percentiles)) between the PJP group (n=78) and *P. jirovecii* colonization group (n=49) were compared using the Mann-Whitney U test, with the statistic denoted as U. qPCR(Ct), polymerase chain reaction cycle threshold; LDH (U/L), lactate dehydrogenase; Hs-CRP (mg/L), hypersensitive C-reactive protein; IL-10 (pg/mL), Interleukin-10; IL-6 (pg/mL), Interleukin-6; IFN-*γ* (pg/mL), interferon-*γ*; IL-2 (pg/mL), Interleukin-2; IL-17A (pg/mL), Interleukin-17A; TNF-*α* (pg/mL), tumor necrosis factor-*α*; IL-4 (pg/mL), Interleukin-4; WBC count (10^9^/L), white blood cell count; Lymphocyte (10^9^/L), lymphocyte; Monocyte (10^9^/L), monocyte; Neutrophil (10^9^/L), neutrophil; NR, normal range; NS, not significant.Significance markers are defined as: ***P<0.001, **P<0.01, *P<0.05

### 3.3 Analysis of mechanistic immune cell subset data

The results of the 17 core model input predictors analysis indicated that PJP patients exhibited an immune state characterized by significantly elevated cytokines (IL-6, IFN-*γ*, IL-10) and a marked reduction in total lymphocytes. To explore the cellular basis of this immunoregulatory imbalance, we performed exploratory analyses on results from regulatory T cell (Treg, defined as CD4^+^CD25^+^FoxP3^+^) testing performed on peripheral blood samples from a subset of 41 patients (26 PJP, 15 P*. jirovecii* colonization), and immune cell subset phenotyping from another subset of 24 patients (17 PJP, 7 P*. jirovecii* colonization). The baseline characteristics (age, sex, underlying diseases) of these subsets showed no statistically significant differences compared with the overall cohort or with the remaining patients not included in these subsets, confirming their representativeness in **Table 4**.

**Table 4:** Baseline characteristic comparisons of patient subsets and the overall cohort in Pneumocystis jirovecii-associated analyses.

Variables	P-value of group 1	P-value of group 2	P-value of group 3	Sig.
Age (years)	0.7655	0.7261	0.1263	NS
Male	1	0.1265	0.3801	NS
Smoking history (⩾ 5 years)	0.3139	0.3864	0.63	NS
Drinking history (⩾ 5 years)	0.3461	1	0.9079	NS
Hypertension	1	0.3926	0.3882	NS
Diabetes mellitus	1	1	0.6665	NS
Tuberculosis	1	0.1937	0.2801	NS
Chronic kidney disease	1	1	0.8084	NS
COPD	0.3994	0.6466	0.7302	NS
30-day mortality rate	0.2779	1	0.2803	NS
Autoimmune disease	0.5244	0.5072	0.7776	NS

This table presents the statistical P-values for comparisons of clinical baseline characteristics across three groups. Regulatory T cells (Treg, defined as CD4^+^CD25^+^FoxP3^+^) were analysed in peripheral blood samples from all 41 patients; immune cell subsets were analysed in a subset of 24 patients (17 with *P. jirovecii* pneumonia (PJP), 7 with *P. jirovecii* colonization). Group 1: comparison of baseline characteristics between the Treg-analysed subset and the overall cohort; Group 2: comparison of baseline characteristics between the immune cell subset-analysed cohort and the overall cohort; Group 3: comparison of baseline characteristics between the combined Treg and immune cell subset-analysed patients and the remaining patients from the overall cohort not included in these two subsets. COPD, chronic obstructive pulmonary disease; NS, not significant (*P >* 0.05). No statistically significant differences were observed in all baseline characteristics across the three comparison groups, confirming the representative baseline distribution of the two analysed subsets relative to the overall cohort

Analysis of flow cytometry results (**Table 5**) showed that the absolute count of Tregs was significantly lower in PJP patients (15.1 vs. 36.9 cells/*µ*L; *P* = 0.016). Furthermore, compared to the *P. jirovecii* colonization group, PJP patients exhibited significantly reduced total lymphocyte counts and related T-cell counts. Total lymphocyte count (670 vs. 1230 cells/*µ*L; *P* = 0.0222), CD3^+^ T cells (461.8 vs. 1097.2 cells/*µ*L; *P* = 0.0158), and CD3^+^CD4^+^ helper T cells (150.6 vs. 498.1 cells/*µ*L; *P* = 0.0133) were all significantly lower in the PJP group. In contrast, no statistically significant differences (*P>*0.05) were observed between the groups for CD3^+^CD8^+^ cytotoxic T cells, natural killer cells (CD3^−^CD56^+^), B cells (CD19^+^), or their respective percentages among total lymphocytes. The CD4^+^/CD8^+^ ratio also did not differ significantly.

**Table 5:** Intergroup comparison of lymphocyte subset indices between the PJP and *P. jirovecii* colonization patients.

Analysis subset	Variables	PJP	*P. jirovecii* colonization	P value	Sig.
subsets of immune cell populations (N = 24)	Lymphocyte count	670 (590–840)	1230 (1130–1780)	0.0222	*
	CD3+	461.8 (277.8–564.5)	1097.2 (776.05–1435.25)	0.0158	*
	CD3+CD4+	150.6 (60.5–339.1)	498.1 (344.3–649.05)	0.0133	*
	CD3+CD8+	183.7 (116.2–366.5)	380.5 (264.95–749.2)	0.1123	NS
	CD4+/CD8+	1.07 (0.8–1.59)	2 (0.915–2.21)	0.3407	NS
	CD3+%	67.2 (52.5–73.7) 23.9	69.4 (63.75–80.6)	0.5253	NS
	CD3+CD4+%	(9–42.8)	36.7 (32.65–43.95)	0.409	NS
	CD3+CD8+%	26.1 (15.7–44.8)	21.5 (18.6–41)	0.7995	NS
	CD3-CD56+	120.2 (34.1–159.1)	152.5 (74.65–220.75)	0.3095	NS
	CD3-CD56+%	13.9 (4.4–23.3)	8.4 (5.25–15.95)	0.4651	NS
	CD19+	70.6 (9.6–166.2)	87.3 (37.6–228.9)	0.6791	NS
	CD19+%	11.2 (2–24.5)	9.2 (2.95–21.9)	0.8736	NS
Treg subset (N = 41)	CD4+CD25+CD127dim	15.1 (10.175–21.925)	36.9 (18.85–60.15)	0.016	*

PJP, Pneumocystis jirovecii pneumonia; Treg, regulatory T cell; CD, cluster of differentiation; NS, not significant.Data are presented as median (interquartile range, IQR). Immune cell subset analysis: A total of 24 patients were included in this subset, consisting of 17 patients with PJP infection and 7 patients with*P. jirovecii* colonization. Treg subset analysis: A total of 41 patients were included in this subset, consisting of 26 patients with PJP infection and 15 patients with *P. jirovecii* colonization. Statistical comparisons between infection and colonization groups within each subset were performed using the Mann-Whitney U test. *P<0.05; NS, not significant (*P*≥0.05).

In summary, these results indicate that, compared to *P. jirovecii* colonization, PJP is associated with lymphopenia, particularly characterized by a significant decrease in the absolute numbers of CD4^+^ T cells and their subsets, including helper T cells and Tregs. These alterations in immune cell populations—particularly the marked reduction in CD4^+^ T cells and Tregs—may reflect an infection-associated state of immune dysfunction.

### 3.4 Construction and validation of the random forest model for differentiating *P. jirovecii* colonization from PJP

The 17 predictor variables (detailed in the Methods), obtained from all 127 patients in the core cohort, were used for model construction. Prior to model development, the raw data of all 127 patients were randomly split into a training set (N = 90) and a validation set (N = 37) at a ratio of 7:3. To ensure intergroup comparability, the proportion of infected cases was 61.1% in the training set and 62.1% in the validation set, representing a well-balanced distribution between the two cohorts. All subsequent feature selection and hyperparameter optimization procedures were performed exclusively on the training set; the validation set was not involved in any model-building decisions until the final model was established, thus guaranteeing an unbiased assessment of model generalization performance.

A random forest classification model was constructed using the randomForest package in R Studio, a baseline model was built with the native default parameters of the package: the number of candidate variables randomly selected at each node split (mtry) was set to 4, a value derived from the default formula for classification tasks (p=17 in this study, with $\sqrt{17}$ integer 4), and the number of decision trees (ntree) was set to the default value of 500. The baseline model yielded an area under the receiver operating characteristic curve (AUC) of 0.899 and an accuracy of 0.856 on the training set, and an AUC of 0.846 (95% confidence interval, CI: 0.703–0.990) and an accuracy of 0.784 on the validation set. This baseline model served as a reference for evaluating the efficacy of subsequent hyperparameter tuning.

**Figure 3:**
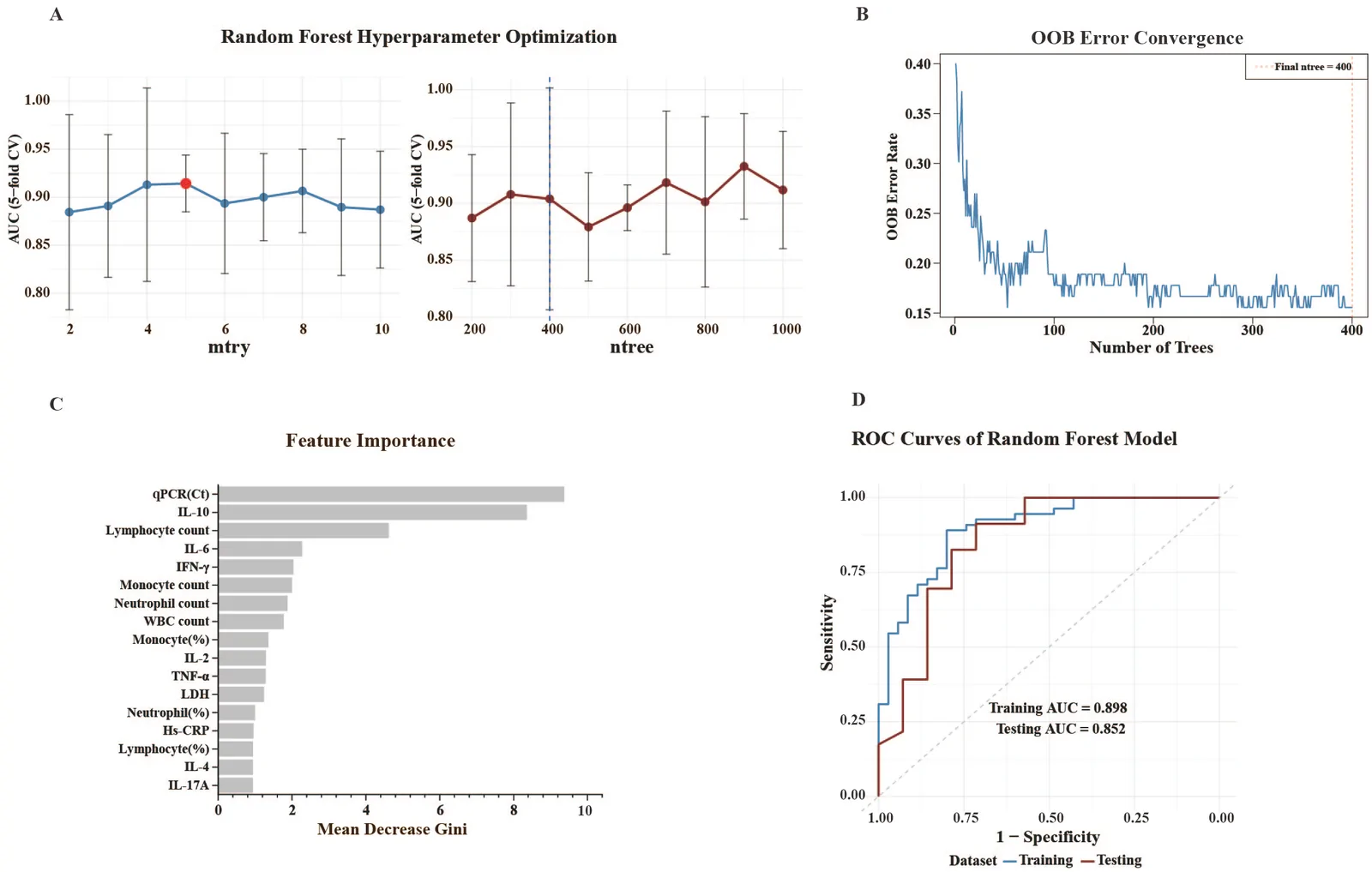
Hyperparameter optimization, performance evaluation and feature importance analysis of the random forest model for distinguishing *P. jirovecii* colonization from infection. **(A)** Five-fold cross-validation AUC values across candidate mtry and ntree values, identifying mtry=5 and ntree=400 as the selected hyperparameters. **(B)** Convergence of the out-of-bag error rate with increasing numbers of trees, with the final ntree set to 400. **(C)** Feature-importance ranking quantified by mean decrease in Gini. **(D)** ROC curves for the training and validation datasets (AUC 0.898 and 0.852, respectively).

To enhance the discriminative performance of the model and mitigate the risk of overfitting, a two-stage grid search for the hyperparameters mtry and ntree was conducted on the training set using 5-fold cross-validation. By comparing the out-of-bag (OOB) error and 5-fold cross-validation AUC across different mtry values (**Figure 3A**), mtry=5 was identified as the optimal parameter, as it maintained a low error rate across all values of ntree. With mtry=5 fixed, we investigated the trend of model error with increasing ntree; the results showed that the OOB error stabilized and reached its minimum when ntree increased to 400, at which point the model performance entered a plateau phase. Considering both predictive accuracy and computational efficiency, the final random forest model was constructed with mtry=5 and ntree=400 to ensure the robustness of the results (**Figure 3B**).

The performance metrics of the optimized model were as follows (**Table 6**): an AUC of 0.898 and an accuracy of 0.8444 on the training set; an AUC of 0.852 (95% CI: 0.709–0.996) and an accuracy of 0.784 on the validation set, with a sensitivity of 0.913 (**Figure 3D**), specificity of 0.571, positive predictive value (PPV) of 0.778, negative predictive value (NPV) of 0.8, and F1 score of 0.84.

**Table 6:** Comparison of the random forest model before and after hyperparameter optimization.

Dataset/parameter group	Performance metrics	Pre-optimization model	Post-optimization model
Training set (N = 90)	AUC	0.899	0.898
	Accuracy	0.856	0.844
Test set (N = 37)	AUC (95% CI)	0.846 (0.703-0.990)	0.852 (0.709-0.996)
	Accuracy	0.784	0.784
Model Parameters	mtry	4 (default)	5 (optimized)
	ntree	500 (default)	400 (optimized)

Performance metrics for the random forest model on the training (N = 90) and test (N = 37) sets are shown before (default: mtry=4, ntree=500) and after (optimized: mtry=5, ntree=400) hyperparameter tuning. Prior to optimization, a discrepancy was observed between training (AUC = 0.899) and test (AUC = 0.846) performance. Post-optimization, this gap narrowed (training AUC = 0.898 vs. test AUC = 0.852), with the performance difference decreasing from 0.053 to 0.046, indicating reduced overfitting and enhanced model generalizability. Values in parentheses represent the 95% confidence intervals for test set AUC.

Assessment of the importance of the 17 predictors in the random forest model, as quantified by the Mean Decrease in Gini coefficient, revealed marked disparities in the contributions of individual variables to differentiating PJP infection from *P. jirovecii* colonization (**Figure 3C**). Core predictors: Pathogen load, evaluated by the qPCR (Ct) value, was the most robust predictor for distinguishing infection from *P. jirovecii* colonization (Mean Decrease in Gini: 9.37), a value considerably higher than those of all other metrics. It was closely followed by the anti-inflammatory cytokine IL-10 (Mean Decrease in Gini: 8.35), indicative of its pivotal role in the immunomodulation of PJP infection.Secondary contributors: A panel of indices reflecting host immune status formed the second tier of predictive factors, including absolute lymphocyte count (4.61), IL-6 (2.26), IFN-*γ* (2.03), absolute monocyte count (1.99) and absolute neutrophil count (1.87).Low contributors: Several routine clinical metrics exerted only limited predictive value. Among these, white blood cell count (1.77), TNF-*α* (1.28), IL-2 (1.28), LDH (1.24) and monocyte percentage (1.35) displayed moderately low importance. In contrast, lymphocyte percentage (0.93), neutrophil percentage (0.99), hsCRP (0.95), IL-17A (0.93) and IL-4 (0.93) exhibited the lowest predictive importance, with all their Mean Decrease in Gini values below 1.0.

## 4 Discussion

Evidence demonstrates that infections by distinct pathogens elicit specific hostimmune responses. Profiling these responses enables high-precision differentiation of the causative pathogen (15), offering a novel diagnostic avenue for PJP.

This study systematically compared the immunological profiles of patients with PJP versus *P. jirovecii* colonization by integrating the 17 core model input predictors (pathogen load and host immune response indicators), leading to the construction of a discriminative diagnostic model based on a Random Forest algorithm. Our central finding is that PJP patients exhibit a state of “hyperinflammation with concomitant immune impairment”, characterized by an increased pathogen burden, elevated levels of pro- and antiinflammatory cytokines (IFN-*γ*, IL-6, IL-10), and concomitant systemic lymphopenia—marked notably by a reduction in the absolute numbers of CD4^+^ T cells and their subsets, including helper Tregs. Prior research has reported relatively high IFN-*γ* levels in PJP patients (16), which aligns with our finding of elevated IFN-*γ* in the PJP group. This may be linked to IFN-*γ*’s potential role in aiding immune evasion of *P.jirovecii* by suppressing macrophage phagocytosis (17). IL-6, a key mediator of the acute-phase response, plays a significant role in pulmonary inflammation triggered by pathogens (18, 19). It has been identified as a predictor for various pulmonary inflammatory conditions and an important prognostic marker in PJP (20, 21). In our study, the significant rise in IL-6 compared to the *P. jirovecii* colonization group likely corresponds to the severe systemic inflammation and lung injury associated with PJP. More instructive is the sharp increase in IL-10, typically regarded as an anti-inflammatory or immunoregulatory cytokine (22, 23). In the context of PJP infection, high IL-10 levels may represent a negative feedback mechanism attempting to curb excessive inflammation (24); however, it might also simultaneously suppress effective T-cell responses against the pathogen, correlating with the observed CD4^+^ T cell exhaustion. When CD4^+^ T cell numbers are significantly reduced (e.g., *<*200/*µ*L in HIV patients) or their function is impaired (e.g., by immunosuppressants post-transplantation), the host fails to effectively clear *P. jirovecii*, facilitating the transition from *P. jirovecii* colonization to PJP (12, 13). In contrast, patients in the *P. jirovecii* colonization group exhibited lower pathogen loads and a more balanced immune state, possibly reflecting a scenario where the immune system successfully controls or even clears the pathogen without inciting excessive tissue damage.

From a translational clinical perspective, while molecular techniques like qPCR and mNGS can detect minute levels of *P. jirovecii* DNA, they cannot discern whether the detected nucleic acids originate from *P. jirovecii* colonization or active infection (25, 26). Our study provides a solution based on objective laboratory parameters to address this core diagnostic challenge in PJP—differentiating active infection from harmless *P. jirovecii* colonization. Combining qPCR (pathogen burden) with host-response markers (such as IL-10 and absolute lymphocyte count) from our set of 17 predictors significantly enhanced discriminatory power. The robust performance of the Random Forest model (AUC = 0.852) validates the potential of this integrated strategy. Furthermore, specific immune signatures, such as marked lymphopenia coupled with high levels of IL-6/IL-10, may not only serve as diagnostic markers but could also correlate with disease severity, laying a foundation for future patient stratification. These findings also point toward potential directions for individualized therapy. For instance, patients exhibiting a pronounced “hyperinflammatory storm” (e.g., extremely high IL-6) might derive clearer benefit from adjunctive corticosteroid therapy, whereas future treatment strategies for patients characterized by severe T-cell exhaustion may need to explore avenues for immune potentiation or reconstitution.

Several limitations of this study should be acknowledged. First, the single-centre retrospective design has inherent limitations. The sample size, particularly for the subsets undergoing in-depth immunophenotyping, was limited, and the availability of data for advanced immunological analyses (Treg and immunophenotyping) was contingent upon whether these tests had been previously ordered in clinical practice, leading to the formation of smaller convenience-based subgroups that may introduce selection bias. However, as shown in **Table 4** the baseline characteristics of these subgroups were comparable to those of the entire cohort. Second, the measurements of cytokines and immune cells were obtained at relatively fixed time points and therefore could not dynamically reflect immune evolution over the disease course. Third, we analysed only seventeen cytokines and did not cover broader immune networks, such as other interleukins or chemokines. Fourth, the lack of direct microscopic confirmation (e.g., by conventional staining methods) for all cases is a limitation inherent to the retrospective design. Although we employed a rigorous, expert-adjudicated composite clinical standard as the reference, future prospective studies incorporating routine cytological or histological confirmation would further strengthen the diagnostic certainty of the classification. Finally,”From a clinical translation perspective, we emphasize that the proposed model is not intended to replace physician judgment or to guide the decision to with hold treatment in patients with high clinical suspicion of PJP. Rather, it is designed as an adjunctive risk stratification tool for challenging scenarios, particularly in patients with indeterminate qPCR results (Ct values in the grey zone of 32–35) or atypical clinical presentations. In such cases, the model’s integrated assessment of pathogen burden and host immune response may provide additional objective evidence to inform the decision to initiate or de-escalate anti-PJP therapy. Furthermore, the identification of distinct immune phenotypes—ranging from hyperinflammatory to severely immunosuppressed—may guide individualized adjunctive therapies, such as corticosteroid use or immune reconstitution strategies, in future studies.

Future research should validate these findings in larger, prospective, multicenter cohorts. Subsequent efforts should focus on: 1) incorporating a more comprehensive panel of immune markers (including other cytokines) to map the immune landscape more finely; 2) conducting longitudinal studies to monitor changes in immune parameters pre- and post-treatment, identifying dynamic biomarkers associated with therapeutic response and prognosis; and 3) exploring the integration of our model with clinical scoring systems to develop user-friendly diagnostic and risk-prediction tools for clinical practice. By deepening our understanding of host-pathogen interactions, we can ultimately advance toward the precise diagnosis and individualized management of PJP.

## Data Availability

The original contributions presented in the study are included in the article/supplementary material. Further inquiries can be directed to the corresponding author.
